# Detection and Molecular Identification of *Salmonella* Virulence Genes in Livestock Production Systems in South Africa

**DOI:** 10.3390/pathogens8030124

**Published:** 2019-08-09

**Authors:** Thobeka P. Mthembu, Oliver T. Zishiri, Mohamed E. El Zowalaty

**Affiliations:** 1 Discipline of Genetics, School of Life Sciences, University of KwaZulu-Natal, Private Bag X54001, Durban 4000, South Africa; 2 Virology and Microbiology Research Group, College of Pharmacy, City University College of Ajman, Al Tallah 2, Ajman, P.O. Box 18484, UAE

**Keywords:** *Salmonella*, zoonosis, pathogenicity, virulence, food-borne, livestock, Xylose-Lysine-Deoxycholate, pathogen, PCR, integron, infection, humans, one-health

## Abstract

Livestock are an important source of protein and food for humans, however opportunistic pathogens such as *Salmonella* spp. turn livestock into vehicles of foodborne diseases. This study investigated the prevalence of virulence genes in *Salmonella* spp. isolated from livestock production systems in two provinces of South Africa. During the period from May to August, 2018, a total of 361 faecal (189), oral (100), environmental (soil (36) and water (27)) and feed (9) samples were randomly collected from different animals (cattle, sheep, goats, pigs, ducks and chickens) that were housed in small-scale livestock production systems from Eastern Cape and KwaZulu-Natal Provinces in South Africa. *Salmonella* spp. were isolated and identified using microbiological and DNA molecular methods. *Salmonella* spp. were present in 29.0% of the samples of which 30.2% belonged to the *Salmonella enterica* species as confirmed by the positive amplification of the species specific *iroB* gene. Virulence genes that were screened from livestock-associated *Salmonella* were *invA*, *iroB*, *spiC*, *pipD* and *int1*. Statistically significant associations (*p* < 0.05) were established between the virulence genes, sampling location, animal host as well as the season when samples were collected. Furthermore, statistically significant (*p* < 0.05) positive correlations were observed between most of the virulence genes investigated. This is one of the recent studies to detect and investigate livestock-associated *Salmonella* spp. in South Africa. This study highlights the importance of continuous monitoring and surveillance for pathogenic salmonellae. It also demonstrated the detection and prevalence of virulent *Salmonella* spp. harbored by livestock in South Africa. This study demonstrated the potential risks of pathogenic *Salmonella enterica* to cause foodborne diseases and zoonotic infections from farm-to-fork continuum using the global one-health approach.

## 1. Introduction

Salmonellae are facultative intracellular Gram-negative bacteria that cause high morbidity and mortality in a wide range of hosts including humans, birds, mammals, and insects [1]. Salmonellae are one of the most problematic, foodborne, and zoonotic pathogens that cause health threats and challenges to general human well-being [2]. *Salmonella* spp. reside in the gastrointestinal tract of warm-blooded animals. The bacteria cause salmonellosis in humans, a disease that is presented mostly by mild diarrhea, also well-known as food poisoning [3,4]. Salmonellosis may be fatal, depending on the dose of infection and the immune status of the infected individual [5]. In the United States, *Salmonella* spp. are currently on the top of the list of pathogens that cause foodborne infections [6]. In South Africa, *Salmonella* spp. are regarded as one of the leading causes of foodborne outbreaks [7]. Foodborne outbreaks were reported in South Africa due to consumption of animal and poultry contaminated products [8,9,10,11]. *Salmonella* infection causes economic losses in the agriculture sector and it negatively impacts food animals which are reared for the generation of income [12].

Although *Salmonella* is a major cause of foodborne diseases in South Africa, there are limited data on the disease since it usually causes self-limiting gastroenteritis and cases are rarely reported [8,12]. It has been reported that six out of seven *Salmonella enterica* serovar Enteritidis outbreaks that occurred in South Africa from 2013 to 2015 were of food origin [13]. It was reported that 141 (43%) out of the 327 foodborne outbreaks reported in South Africa between 2013 to 2017 were reported in warmer months from KwaZulu-Natal [7]. An outbreak of food origin caused by *S. enterica* serotype Virchow was reported at a school in South Africa [14].

Surveillance of *Salmonella* is frequently conducted by different organizations worldwide in order to study its prevalence and epidemiology [8,15]. In South Africa surveillance is mainly the responsibility of governmental departments such as the Department of Health as well as the Department of Agriculture, Forestry and Fisheries. In order to bolster the surveillance, government departments have collaborations with local universities and other research institutions. *Salmonella* species play a role in metabolism when it is in a non-virulent state [16]. Factors such as stressful conditions, environmental changes and mutations can trigger virulence in a bacterium thus turning a previously non-virulent strain into a pathogenic strain [17].

The *Salmonella* genus includes more than 2500 serological variants (serovars) and is broadly categorized into *S*. *bongori* and *S*. *enterica* species [18]. According to the U.S. Centers for Disease Control (CDC) *S. enterica* is further subdivided into six subspecies that are designated by taxonomic names such as *S. enterica* subsp. *enterica*, *S. enterica* subsp. *salamae*, *S. enterica* subsp. *arizonae*, *S. enterica* subsp. *diarizonae*, *S. enterica* subsp. *houtenae* and *S. enterica* subsp. *indica*. *S. enterica* species is highly diverse consisting of more than 2600 serovars which are further divided into typhoidal *Salmonella* and non-typhoidal *Salmonella* (NTS), depending on the disease it causes [19,20]. Typhoidal *Salmonella* spp. are restricted to human hosts while non-typhoidal *Salmonella* spp. can infect a wide range of hosts [21]. Faecal shedding of NTS results in environmental contamination and transmission to humans, leading to disease outbreak [16]. NTS has a broad host range and is often associated with foodborne outbreaks in humans [21]. *S. enterica* serovar Typhimurium and *S. enterica* serovar Enteritidis are the most frequently reported pathogens in *Salmonella* outbreaks and the prominent cause of gastroenteritis in humans [22,23].

*Salmonella* pathogenicity is mediated by numerous genes such as *invA*, *spiC* and *pipD,* which code for effectors that induce successful host infection. Pathogenicity of *Salmonella* is expressed in three ways such as host cell invasion, intracellular survival and colonization [24]. Numerous virulence genes are essential for *Salmonella* pathogenesis and these genes are located on various elements of the genome including the chromosome, plasmids, integrated bacteriophage DNA, *Salmonella* pathogenicity islands (SPIs), and *Salmonella* genomic islands (SGIs) [19,25]. SPIs are large gene cassettes and only SPI-1 and SPI-2 (not all SPIs) encode a membrane-associated type III secretion system (T3SS) [26] which secretes a pool of 44 effector proteins [27], that alter the functioning of eukaryotic cells in order to facilitate bacterial pathogenicity inside the cell [28,29,30]. Previous studies reported that SPIs are acquired by horizontal transmission and vertically pass to new clones [31]. More than 20 SPIs have been characterized, with greater focus on SPI-1 and SPI-2 that function via encoded T3SS since they harbor host invasion and intracellular survival genes [29,32]. Inside the host cell, SPI-2 expresses genes that are important in intracellular survival, proliferation, and persistence in internal organs such as the spleen and liver [30,33]. *Salmonella* spp. use virulence genes and factors located in SPI-1 for cell invasion and to initiate its pathogenicity [29]. The invasion A (*invA*) is one of the most studied virulence factors that is also used as a biomarker for *Salmonella* spp. detection as it contains sequences that are unique to the genus *Salmonella*. [34] Invasion A is a factor in the outer membrane of *Salmonella* spp. that is responsible for entering the host epithelial cells in the intestines thus initiating infection [34]. The *inv* locus in *S. enterica* serovar Typhimurium was characterized and it was reported that *invA* is essential in the display of virulence in the intestine [35].

One of the most important genes is *iroB*, a Fur-regulated gene located in a large DNA region which is used in the detection of *S. enterica* subspecies *enterica* [36,37]. Previous studies which detected typhoid and non-typhoid *Salmonella* by PCR used *invA* and *iroB* together with flagellar genes [38,39]. Furthermore, *iroB* was used to detect *Salmonella* from blood in another study [40]. The *IroB* gene is a member of the *iroA* (*iroBCDEN*) gene cluster which is responsible for the synthesis and transport of enterobactin, a siderophore produced by *Salmonella* spp. and is essential for iron uptake inside the host [41]. Besides enabling bacterial iron uptake, expression of the *iroA* cluster also facilitates the host immune escape by interrupting macrophage homeostasis [42]. The specific role of *iroB* is to encode glucosyltransferase which glucosylates enterobactin [41]. Enterobactin glucosylation contributes to the virulence of the bacteria by preventing the host antimicrobial protein (lipocalin-2) from sequestering the siderophore [43,44].

*spiC* is another gene in the SPI-2 that is essential for intracellular survival and host defense escape [45]. Macrophages are important innate immune barriers which defend the host against infections and their function is activated by gamma interferon and facilitated by factors such as cytokines and eicosanoids [46]. Upon activation, macrophages kill pathogens that are capable of surviving inside them. In order to escape the host’s defense, *spiC* is involved in the signal transduction pathway which expresses the suppressor of cytokine signaling, leading to gamma interferon signaling inhibition [45]. It was reported that *spiC* is also involved in the translocation of effectors into the cytosol of macrophages [47].

The SPI-5 harbors six genes in which mutations in four of these genes were reported to radically lower enteropathogenicity [48,49]. *pipD* is one of the genes in the SPI-5 that is involved in inflammatory enteritis by coding a cysteine protease homolog that is essential in the long term systemic infection [48,49,50].

Gastroenteritis is the most common disease caused by non-typhoidal *Salmonella*. This disease usually resolves without treatment but it can be systemic in severe cases and require antimicrobial treatment. There is, however, an enormous challenge with using antibiotics as *Salmonella* is one of the ‘superbugs’ which are resistant to several classes of antibiotics [51]. The antimicrobial resistance phenotype is attributed to the possession of class 1 integron by some of the *Salmonella* serovars.

The class 1 integron is a mobilizable cluster of antimicrobial resistance genes found in *Salmonella* genomic island [52,53,54,55]. Class 1 integrons are made up of integrase gene, a primary recombination site and a promoter region [56]. The role of *int1* is to recombine gene cassettes (associated with antibiotic resistance), which are only transcribed in an integron since they lack a promoter [57,58]. Class 1 integron carries gene cassettes for resistance to antibiotics such as those which were used as first line treatment for salmonellosis. The presence of class 1 integrons carrying gene cassettes in virulent *Salmonella* spp. increases the threat to humans as it limits the treatment options available [59,60]. Infections by non-tyhpoidal *Salmonella* spp. affect both developing and developed countries. Studies and incidences revealed that food animals are the carriers of NTS and are potential zoonotic sources of infection to humans [61,62,63]. Against this background, this study focused on the detection and determination of the prevalence of virulent *Salmonella* spp. in livestock production systems in the KwaZulu-Natal and Eastern Cape Provinces in South Africa.

## 2. Materials and Methods

### 2.1. Ethical Approval

The study was approved by the Animal Research Ethics Committee of the University of Kwa-Zulu Natal (Reference numbers AREC/051/017M, AREC 071/017 and AREC 014/018). The field sampling protocols, samples collected from animals, and the research were conducted in full compliance with Section 20 of the Animal Diseases Act of 1984 (Act No 35 of 1984) and were approved by the South African Department of Agriculture, Forestry and Fisheries DAFF (Section 20 approval reference number 12/11/1/5 granted to Prof. Dr. ME El Zowalaty).

### 2.2. Samples and Pre-Enrichment

**Figure 1:**
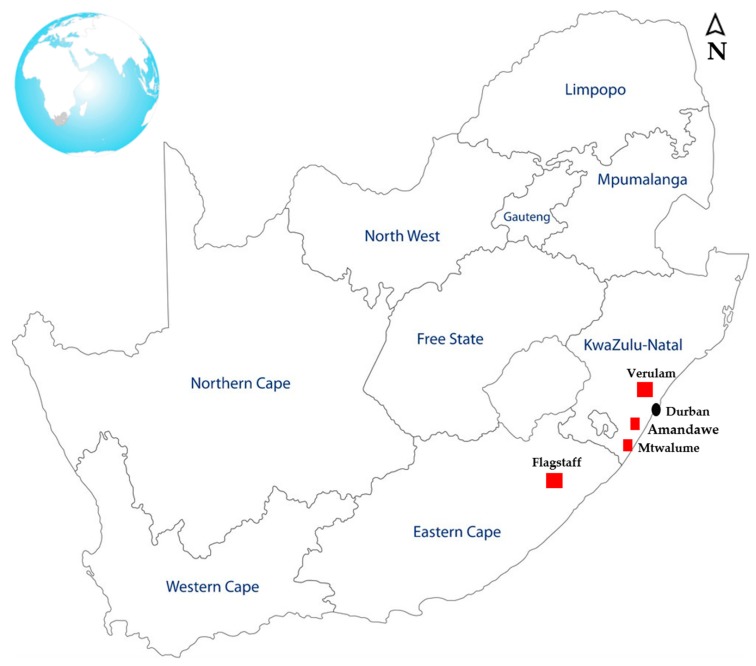
Map of South Africa showing the geographic locations of the farms where samples were collected for this study.

During the autumn and winter months of the year 2018, a total of three hundred and sixty-one (361) oral, faecal, soil, water and feed samples were randomly collected from different animal hosts such as cattle, sheep, goats, pigs, ducks and chickens. The animals were housed in small-scale commercial farms in Flagstaff (O.R Tambo, Eastern Cape), Verulam (eThekwini, KwaZulu-Natal) and the South Coast (Amandawe and Mtwalume, UGU, KwaZulu-Natal) as depicted in Figure 1.

All samples were randomly collected between May and August 2018 from different farms in Eastern Cape and KwaZulu-Natal Provinces of South Africa. In the farms, livestock were bought from large-scale farms and were sold to the communities. ISO 6579-1 was used to collect and isolate *Salmonella* however, PCR was used for detection and confirmation of the presence of *Salmonella* spp. Fresh environmental faecal samples emanating from livestock, as well as samples from livestock environments including soil, water and feed were collected using sterile collection swabs. All swab samples were transferred into 10 mL of 0.1% buffered sterilized peptone water (Merck, Johannesburg, South Africa). Water samples were collected from the containers inside the livestock houses. Samples were transported on ice to the discipline of genetics laboratories where enrichment of the samples was conducted by incubation overnight at 37 °C.

### 2.3. Selective Enrichment

From each of the enriched samples, 0.1 mL was aseptically transferred into 10 mL of Rappaport Vassiliadis (RV) broth and incubated for 24 h at 42 °C (Sigma-Aldrich, Mumbai, India). RV is a selective medium that is enriched with malachite green which inhibits the growth of microorganisms other than *Salmonella*. A previously identified and confirmed *S. enterica* was used as a positive control for this experiment [63]. Microbiological isolation was performed on Xylose-Lysine-Deoxycholate (XLD) agar (Sigma-Aldrich, Buchs, Switzerland) by aseptically streaking a loopful of the culture from RV broth onto the XLD plates. *S. enterica* is differentiated from *Escherichia coli* and *Shigella* spp. by producing red colonies with black centers on XLD agar. After 24 h of incubation at 35 °C, the plates were observed for the growth of the expected colonies. Single colonies were picked from each plate and transferred into tubes containing 10 mL of tryptose soy broth (Merck, Johannesburg, South Africa) and incubated at 37 °C for 18–24 h. A 2 mL of the culture was used for DNA extraction. Equal amounts of 0.5 mL each of 60% glycerol and *Salmonella* pure culture were mixed in 1.5 mL cryotubes and stored at −80 °C for future use.

### 2.4. DNA Extraction

Total genomic DNA was extracted from *Salmonella* cultures using the conventional boiling method. One milliliter of the cultured sample was transferred into 1.5 mL Eppendorf tube and centrifuged at 14,000× *g* for 5 min. The supernatant was discarded and another 1 mL of culture was added to the pellet and centrifuged again to get a bigger pellet. Six hundred µL of sterile distilled water was added to the pellet and centrifuged for 5 min at 14,000× *g*. The supernatant was discarded, 200 µL of sterile distilled water was added again and incubated in a heating block at 100 °C (Labnet, FL, USA) for 10 min with immediate cooling on ice for 5 min. After cooling, the sample was centrifuged at 14,000× *g* for 5 min; the resulting supernatant was transferred into a fresh Eppendorf tube and stored at −20 °C until use in PCR.

### 2.5. Molecular Confirmation of Salmonella spp. Using PCR

*Salmonella* spp. isolates were confirmed by amplifying the *invA* gene, which is genus specific and the *iroB* gene for identification of *S. enterica* using specific primers as previously reported [36,64].

The PCR reaction volume was 25 µL which consisted of 12.5 µL *Taq* master mix (Thermo-Fischer Scientific, Johannesburg, South Africa), 0.5 µL each of forward and reverse primers, 6.5 µL sterile distilled water and 5 µL template DNA. The PCR reaction conditions consisted of initial denaturation cycle for 5 min, followed by 34 amplification cycles for *invA* gene using the following conditions: Denaturation for 30 s at 95 °C, annealing for 30 s at 61 °C, extension for 1 min at 72 °C and final extension for 5 min at 72 °C. The same amplification parameters were used for the *iroB* gene using a different annealing temperature of 55 °C. The PCR amplicons were stored at −20 °C till future use.

### 2.6. Gel Electrophoresis and Visualization of PCR Products

One and a half percent (1.5%) agarose gel was prepared by slightly boiling 1.5 g agarose powder (Cleaver scientific, Rugby, UK) in 100 mL of 1× TAE buffer (Bio Concept, Allschwil, Switzerland). A volume of two µL of ethidium bromide was added to the gel before pouring it into the casting tray. A one hundred bp molecular weight maker (New England biolabs, Beijing, China) was used to estimate the size of the products. A volume of two µL of the molecular weight marker was mixed with 2 µL of 6× purple dye and diluted with 8 µL of nuclease free water. A volume of ight µL of each of the PCR products was loaded in each well. Electrophoresis was carried out at 80 volts for 55 min using Enduro gel XL electrophoresis system (Labnet, FL, USA). DNA bands from the gel were visualized using ChemiDoc MP imaging system (Bio-Rad, CA, USA).

### 2.7. Determination of Virulence Profiles of Salmonella Isolates

The presence of virulence genes was determined by detecting *spiC*, *pipD* and *int1* genes using previously reported [65,66]. PCR amplification conditions at different annealing temperatures [65,66].

## 3. Statistical Analyses

Descriptive statistics (IBM SPSS, version 25) and Microsoft excel 2016 were used to determine the frequencies of *Salmonella* spp. in each livestock species. The effects of location (whether the samples were collected from Flagstaff, Verulam or South Coast), animal host (chicken, pig, sheep, cow, duck, and goat) and season of sampling (autumn or winter) on the presence of virulent *Salmonella* spp. were investigated using the Fischer’s exact test. The Fischer’s exact test is a parametric test of significance that is used in the place of a Chi- Square test in two by two tables. The Pearson’s correlation test was implemented in order to establish the strength and direction of the relationship between the virulence genes. Furthermore, binary logistic regression was used to model the association between the binary outcomes (presence or absence of *Salmonella* spp. and virulence genes) and exposure variables (location, animal host, sampling season, sample material). The dependent variable was determined whether a virulence gene was present (1) or absent (0). The association between using XLD and PCR in detecting *Salmonella* spp. was measured via the Pearson’s correlation test. The null hypothesis tested was that there is no significant (*p* > 0.05) association between location, animal host and season of sampling in the prevalence of virulent *Salmonella* spp. The statistical results were regarded as significant only if the *p* < 0.05. All statistical tests were performed using IBM SPSS software (version 25).

## 4. Results

Out of the collected 361 samples (114 samples from chicken, 79 samples from goat, 58 samples from pig, 50 samples from sheep, 50 samples from cow and 10 samples from duck) as shown in Table 1 it was found that 195 (54%) samples showed positive growth on RV medium and XLD agar.

The positive control strain showed red colonies with black centers, as expected for *S. enterica* on XLD agar however, some of the XLD plates with tested samples showed yellow colonies as a result of lactose fermentation, a characteristic used to differentiate *Salmonella* spp. from *E*. *coli* spp. Single colonies were selected for DNA extraction and confirmation by PCR amplification.

**Figure 2:**
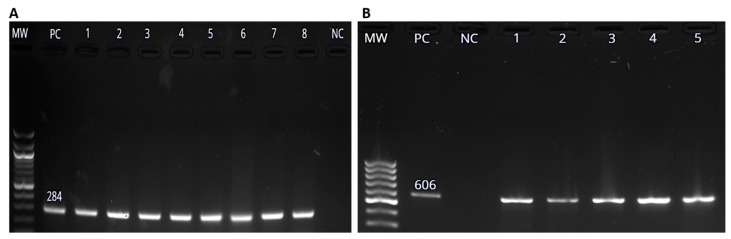
Molecular detection of *Salmonella enterica* using PCR methods. (**A**) *Salmonella* spp. *invA* gene amplicon (284 bp) visualized on 1.5% agarose gel. Lane MW represents the molecular weight marker is the 100 bp DNA ladder (New England Biolabs, Beijing, China). Lane PC represents the positive control and lanes one to eight represents test samples. Lane NC represents the negative control (NC). (**B**) The 606 bp amplicon of *iroB* gene on 1.5% agaroe gel. Lane PC represents the positive control and lanes one to five represent test samples. Lane NC represents the negative control (NC).

The *invA* gene is a genus specific marker that is used for detection of *Salmonella* spp. [64]. Total genomic DNA was extracted from all the 195 isolates which showed positive growth on XLD agar. The extracted DNA was of pure quality, with the mean absorbance ratio (A260/A280) of 2.0, which is accepted for pure DNA. Positive amplification of the *invA* gene was regarded as an indication of the presence of *Salmonella* spp. Out of the 195 presumptive *Salmonella* isolates, 106 (29.4% of the collected samples) were confirmed to be *Salmonella* spp. by amplification of the *invA* gene (Figure 2A). *S. enterica* was confirmed by the detection of the *iroB* gene of band size of 606 bp (Figure 2B) in 32 out of the 106 (30.2%) samples [64].

The total number of samples collected from each animal species were shown in Table 1. The prevalence of *Salmonella* spp. in different hosts was determined and it was found that the highest prevalence was in ducks, followed by chickens, sheep, pigs, cows and goats respectively. In this study, 30.2% of the *Salmonella* isolates were confirmed to be *S. enterica* by *iroB* gene amplification. The 606 bp amplicon of the *iroB* gene was shown in Figure 2B.

**Figure 3:**
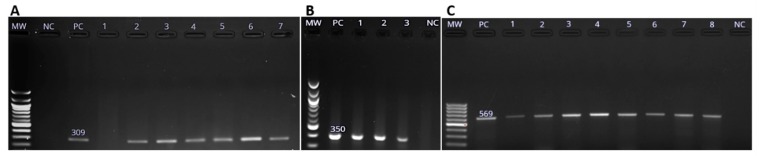
Molecular detection of *Salmonella enterica* virulence genes using PCR methods. (**A**)**.** The 309 bp *spiC* gene amplicon, (**B**) *pipD* gene amplicons (350 bp), and (**C**) The 569 bp amplicon of *int1* gene as visualized on 1.5% agarose gel. Lanes labelled MW, PC and NC are the 100 bp molecular weight marker (New England Biolabs, Beijing, China), positive control (PC) and negative control (NC) respectively. Numbered lanes represent the tested isolates.

Figure 3 show the amplification of virulence genes of *Salmonella* spp, (A) a 309 bp band for *spiC* amplicon, (B) a 350 band for bp *pipD* amplicon and (C) a 569 bp band for *int1* amplicon respectively.

**Figure 4:**
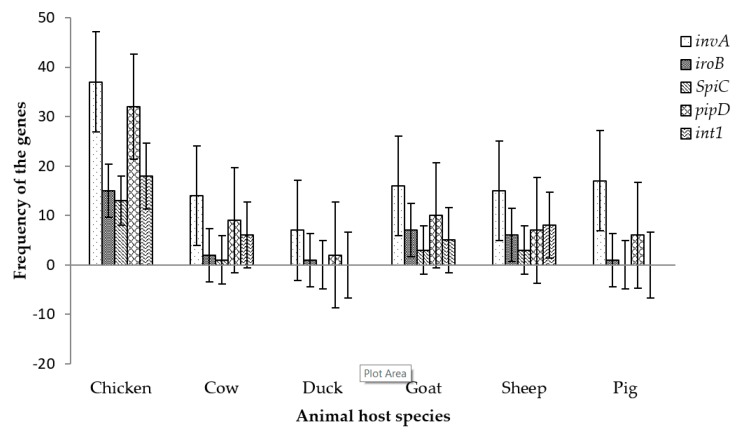
The distribution of *Salmonella* virulence genes in animal host species in this study.

The distribution of the virulence genes in the current study was found to be more frequent in chicken, goat, sheep and cow (Figure 4), with eight chicken isolates, one cow isolate, one sheep isolate and two goat isolates portraying the capability to manifest infection as all isolates from all livestock production animals harbored the screened virulence genes. The frequency of the tested genes was highest in chicken while it was not significantly different in other livestock hosts (Figure 4). However, duck and pig possessed only three of the tested virulence genes respectively. Isolates from goat and sheep had a higher prevalence of virulence genes compared to cow hosts.

**Figure 5:**
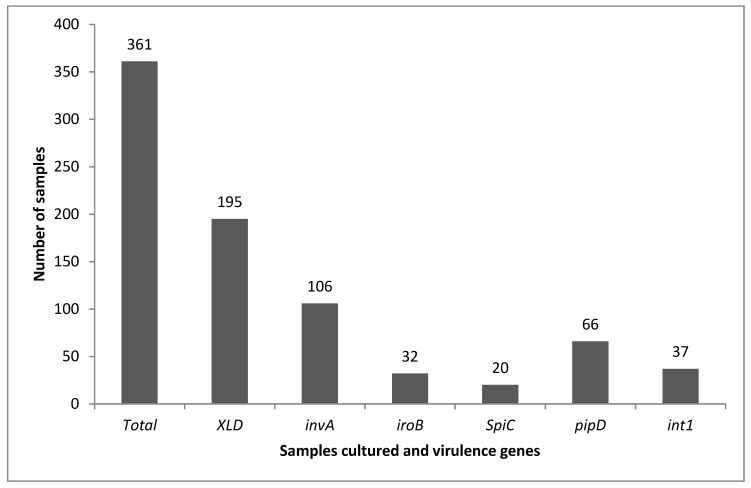
Total number of samples collected and number of positive samples after culturing on Xylose-Lysine-Deoxycholate (XLD) agar and screening of virulence genes.

The overall results on the prevalence of the virulence genes are illustrated in Figure 5. Out of the 106 isolates with *invA* gene, 30% possessed *iroB* gene while 62.3% possessed *pipD* gene, 18.9% possessed *spiC* gene and the *int1* gene was found in 34.9% of the isolates. *PipD* was the most prevalent virulence gene compared to the other tested genes besides *invA*. The difference in the prevalence of the tested virulence genes can be attributed to the location of the gene in *Salmonella*. *SpiC* and *iroB* are both located in the SPI-2 which is found only in *S. enterica* while *invA* and *pipD* are in SPIs 1 and 5. *Int1* is found in the SGI-1 and plasmids.

The effects of location, animal host species and sample material on the presence of *iroB*. *spiC*, *pipD*, and *int1* were evaluated by binary logistic regression. Animal host species and sample material did not significantly (*p* > 0.05) predict the presence of all the virulence genes. Table 2 showed that location significantly (*p* < 0.05) contributes to predicting the presence of *iroB*, *pipD* and *int1*. Verulam and Flagstaff were statistically significant (*p* < 0.05) for location. The odds ratio reveals that Verulam was more likely to have the presence of *iroB* [OR = 5.429 (1.577, 18.686)], *pipD* [OR = 19.991 (2.330, 171.530)] and *int1* [OR = 8.053 (1.801, 59.968)] compared to Flagstaff.

The association between using XLD and amplifying the *invA* gene, a universal marker for *Salmonella* spp., used for *Salmonella* detection was assessed via the Pearson’s correlation following the difference in the results obtained from these methods (Figure 5). A significant (*p* < 0.05) positive 40.2% correlation between using XLD and *invA* amplification was obtained (Table 3). A significant positive correlation (*p* < 0.05) between the tested virulence genes was observed, except for *spiC* and *int1*. The highest correlation was between *spiC* and *iroB* which are both located in the SPI-2. Although the tested virulence genes have different locations; they are all responsible for virulence in *Salmonella* therefore the significant correlation means that one gene can be used to predict the presence of another gene.

The Fisher’s exact test was used to investigate the association between the presence of virulence genes and location, animal species, seasons of sampling as well as sample material. As shown in Table 4, there was a significant (*p* < 0.05) association between the prevalence of the virulence genes with location, animal host and season of sampling however, there was no significant (*p* > 0.05) association between sample material and virulence genes except for *int1*. It was, therefore, evident that the variables that were tested except the sample material significantly influenced the presence or absence of virulence genes.

## 5. Discussion

*S. enterica* is responsible for infections in humans and animals, with serovars Enteritidis and Typhimurium being the most reported [67]. The present study investigated the prevalence and genetic characteristics of *Salmonella* virulence genes in livestock production systems in South Africa using microbiological culturing and molecular methods.

It is recommended that both microbiological culture methods and DNA molecular techniques are concurrently applied for the detection of *Salmonella* spp. even though culturing is more laborious and time consuming while molecular techniques are quick and more sensitive [68,69]. Of interest to this study was that most of the XLD agar plates had yellow colonies instead of the expected red colonies with black centers. Yellow-pigmented colonies appear due to lactose fermentation by the microorganisms. *Escherichia coli* grow as yellow colonies on XLD while *Salmonella* spp. is known as non-lactose fermenters and appear as pink with black center colonies. There is some controversy however, with using XLD for *Salmonella* spp. detection as there are *Salmonella* serovars which have horizontally inherited the lactose fermentation gene from *E. coli* [70]. The acquisition of the *lac* operon by *Salmonella* spp. reduces the virulence potential of the pathogen [71]. It is worth mentioning that microbial culture methods are presumptive and have to be complemented by genomics and molecular methods for the accurate identification and subsequent characterization of microbial species. In the current study, we called both red colonies with black centers as well as yellow colonies because they are both presumptive. The final confirmation was based on molecular PCR methods and the positive amplification of the genus specific biomarker, i.e., the *invA* gene.

Several studies have reported that *S. enterica* serovar Typhimurium and other *S. enterica* serovars which grow as yellow colonies on XLD agar [72,73]. Out of the 195 colonies that grew on the presumptive XLD agar, only 35 were red with black centers while 160 colonies were yellow pigmented, and some with black centers. Screening of the 195 isolates for the *invA* gene showed that the prevalence of *Salmonella* spp. was 29% (106 out of 361 samples) as depicted in Figure 5. This raises a concern regarding the microbiological media which use lactose fermentation characteristic for differentiating *Salmonella* spp. from *E. coli* as some *Salmonella* isolates can be falsely reported as *E. coli* based on phenotypic characters and colony morphology. Our findings revealed that 32 samples (30.2%) of the *Salmonella* isolates were confirmed to be *S. enterica* using molecular PCR methods of *iroB* gene amplification. This could be explained that the 74 *invA* positive-*iroB* negative *Salmonella* isolates may belong to *S. bongori* species and suggested a co-infection which requires further investigation. In addition, as shown in the supplementary data, it may be indicated that the sensitivity of the *iroB* PCR to identify *S. enterica* was not perfect, since five *iroB*-negative samples were *spiC* positive. Since *spiC* is a SPI-2 gene and this island is specific to *S. enterica* (not present in *S. bongori*), this result indicated that at least these five samples also correspond to *S. enterica*.

In the present study, we only focused on the detection of *S. enterica* since this species is of national public health importance and is the main cause of salmonellosis in the area.

Several studies reported the isolation of *Salmonella* spp. from food animals [62,74,75]. Previous studies reported lower prevalence rates of *Salmonella* spp. than the results of the current study with rates of 2.81%, 8.3% and 10.4%, respectively [62,74,75]. However; recent studies reported higher *Salmonella* spp. prevalence rates of 51% and 48%, respectively [63,76]. The difference in the prevalence of *Salmonella* spp. isolated from livestock can be explained by factors such as environmental conditions, farm management, and biosecurity practices. Most of the previous studies focused on *Salmonella* spp. isolation from poultry and poultry products [63,77,78]. Our study is unique in that it determined the prevalence of *Salmonella* spp. in different animals and animal hosts including avian, swine, ovine, and bovine, some of which were housed together within the same epidemiological distance. As previously alluded, *Salmonella* spp. may be in a virulent or non-virulent state and asymptomatic food animals are possible potential sources of transmission of virulent *Salmonella* to humans [16].

Since *Salmonella* pathogenicity is determined by genes which work collaboratively for successful infection, *invA*, *iroB*, *spiC* and *pipD* were screened from the isolates. The possibility of the isolates to be resistant to antibiotics was determined by screening for the class 1 integron gene. Figure 4 shows the prevalence of the virulence genes in all the livestock. Prevalence rates of 47% for *SpiC* and 35% for *pipD* in *Salmonella* spp. isolated from chickens in South Africa was reported [63]. Another study reported prevalence rates of 78% for *spiC* and 95% for *pipD* [79] in clinical samples from human and livestock. The prevalence rates of *spiC* were higher in these studies [63,79] than the rate in the current study however, the prevalence rate of *pipD* in the present study was higher than that was previously reported [63].

*IroB*, *spiC* and *pipD* are located in the SPIs while *int1* (class 1 integron) is located in the *Salmonella* genomic island-1 which can explain the difference in the prevalence of these genes with respect to sample material. SPIs are found in the chromosome of pathogenic strains of *Salmonella* spp. while an integron is a mobile element that can integrate into the chromosome of a bacterium [80,81,82]. The presence of 34.9% *int1* (Figure 5) from isolates in the current study explains that there is a possibility of antibiotic resistance emergence in the isolated *Salmonella* spp. The class 1 integron is associated with resistances to antibiotics such as ampicillin, chloramphenicol, streptomycin, sulfonamides ant tetracycline [83], which were used as first line drugs for the treatment of salmonellosis. The detection of different virulence genes in the *Salmonella* isolates in this study represents a public health threats including zoonotic potential and development of antimicrobial resistance. This study demonstrated that the presence of the virulence genes was significantly (*p* < 0.05) predicted by location when location, animal host species, season of sampling and sample material were evaluated by binary logistic regression as shown in Table 2. Interestingly, the findings of the present study showed that the presence of *int1* gene is more associated to faecal samples, and the presence of virulence genes is more associated to the samples obtained in winter compared to autumn. Environmental conditions in which the animals are housed and reared might have an impact on triggering virulence in *Salmonella*. It was previously reported that the risk of *salmonellosis* is related to an increase in temperature in coastal areas compared to non-coastal areas [84]. Similarly, in the present study, Verulam is an urban area where livestock feed mostly on preserved food, which have traces of chemicals and amino acids while livestock in rural areas feed on natural grass that could explain the high prevalence of virulent *Salmonella* spp. in Verulam.

## 6. Conclusions

The findings of the current study showed that food animals are a potential source of virulent *Salmonella* spp., exposing humans to zoonotic infections through potential exposure route via food or direct exposure. Control and biosecurity measures are not always implemented in small-scale farms, particularly in rural areas in South Africa. Small-scale chicken farms are abundant in South Africa, as they do not require large hectares of land and chicken are more feasible and economic to rear compared to the other livestock animals. *Salmonella* spp. carried by livestock in this study does not only pose infection risks to humans but also has the potential of being resistant to antibiotics once infection has initiated and manifested. Some people in rural areas lack knowledge about contamination risk that comes with livestock therefore action plans must be taken to educate people about the importance of hygiene, especially with animal and food handling. This study also demonstrated the importance of implementing one-health control measures in addressing the challenges of foodborne disease and virulence in zoonotic pathogens including *Salmonella* spp.

## Figures and Tables

**Table 1:** Number of samples collected from livestock farms in Flagstaff, Verulam and South Coast in South Africa in 2018.

Animal Host	Flagstaff					Verulam					South Coast					Total	Positive Samples (%)
	Oral	Fecal	Feed	Soil	Water	Oral	Fecal	Feed	Soil	Water	Oral	Fecal	Feed	Soil	Water		
Chicken	0	0	0	0	0	0	24	0	0	0	40	40	0	5	5	114	10.25
Ducks	0	10	0	0	0	0	0	0	0	0	0	0	0	0	0	10	1.94
Cow	0	5	0	5	0	0	20	0	0	0	0	10	0	5	5	50	3.88
Goats	10	9	0	6	6	0	10	0	0	0	17	16	0	0	5	79	4.43
Sheep	12	10	0	6	0	0	10	0	0	0	4	8	0	0	0	50	4.15
Pigs	17	17	9	9	6	0	0	0	0	0	0	0	0	0	0	58	4.71
Total	39	51	9	26	12	0	64	0	0	0	61	74	0	10	15	361	29.36

**Table 2:** The effect of location as a predictor for the presence *iroB*, *pipD* and *int1* as measured by the binary logistic regression.

	*p*-Value	Odds Ratio	95% C.I. for Odds Ratio
*iroB*			
Location	0.005		
Verulam	0.007	5.429	(1.577, 18.686)
South coast	0.881	0.924	(0.330, 2.587)
*pipD*			
Location	0.007		
Verulam	0.006	19.991	(2.330, 171.530)
South coast	0.994	0.994	(0.237, 4.173)
*Int1*			
Location	0.022		
Verulam	0.042	8.053	(1.801, 59.968)
South coast	0.409	0.564	(0.145, 2.193)

**Table 3:** Pearson’s correlation analysis measuring the strengths of the relationships between the virulence genes and between using XLD and *invA* for the detection *Salmonella* spp.

Variables	Pearson’s Correlation (*p*-Value)
XLD and *invA*	0.402 (0.000)
*iroB* and *spiC*	0.407 (0.000)
*iroB* and *pipD*	0.258 (0.008)
*iroB* and *int1*	0.294 (0.002)
*spiC* and *pipD*	0.357 (0.000)
*spiC* and *int1*	0.102 (0.298) *
*pipD* and *int1*	0.325 (0.001)

* The correlation is not significant as *p* > 0.05.

**Table 4:** The *p*-values obtained from Fischer’s exact test investigating the association between the variables (location, animal host, sampling season, sample material) and the virulence genes.

Variable	*iroB*	*spiC*	*pipD*	*Int1*
Location	0.002	0.000	0.000	0.000
Animal host	0.037	0.019	0.002	0.000
Sampling season	0.001	0.000	0.000	0.000
Sample material	0.345 *	0.467 *	0.365 *	0.004

* There is no significant association as the *p* > 0.05.

## Supplementary Materials

Supplementary File 1
